# Inhibition of Akt/mTOR/p70S6K Signaling Activity With Huangkui Capsule Alleviates the Early Glomerular Pathological Changes in Diabetic Nephropathy

**DOI:** 10.3389/fphar.2018.00443

**Published:** 2018-05-23

**Authors:** Wei Wu, Wei Hu, Wen-Bei Han, Ying-Lu Liu, Yue Tu, Hai-Ming Yang, Qi-Jun Fang, Mo-Yi Zhou, Zi-Yue Wan, Ren-Mao Tang, Hai-Tao Tang, Yi-Gang Wan

**Affiliations:** ^1^Department of Traditional Chinese Medicine, Nanjing Drum Tower Hospital Clinical College of Traditional Chinese and Western Medicine, Nanjing University of Chinese Medicine, Nanjng, China; ^2^Department of Traditional Chinese Medicine, Nanjing Drum Tower Hospital, The Affiliated Hospital of Nanjing University Medical School, Nanjing, China; ^3^Department of Pharmacy, Nanjing Drum Tower Hospital, The Affiliated Hospital of Nanjing University Medical School, Nanjing, China; ^4^Department of TCM Health Preservation, Second Clinic Medical School, Nanjing University of Chinese Medicine, Nanjing, China; ^5^Department of Social Work, Meiji Gakuin University, Tokyo, Japan; ^6^Institute of Huanghui, Suzhong Pharmaceutical Group Co., Ltd., Taizhou, China

**Keywords:** Huangkui capsule, hyperoside, diabetic nephropathy, glomerular hypertrophy, glomerular basement membrane thickening, mesangial expansion, Akt/mTOR/p70S6K signaling pathway

## Abstract

Huangkui capsule (HKC), a Chinese modern patent medicine extracted from *Abelmoschus manihot* (L.) medic, has been widely applied to clinical therapy in the early diabetic nephropathy (DN) patients. However, it remains elusive whether HKC can ameliorate the inchoate glomerular injuries in hyperglycemia. Recently the activation of phosphatidylinositol-3-kinase (PI3K)/serine-threonine kinase (Akt)/mammalian target of rapamycin (mTOR) signaling and its downstream regulator, 70-kDa ribosomal protein S6 kinase (p70S6K), play important roles in the early glomerular pathological changes of DN including glomerular hypertrophy, glomerular basement membrane (GBM) thickening and mild mesangial expansion. This study thereby aimed to clarify therapeutic effects of HKC during the initial phase of DN and its underlying mechanisms. Fifteen rats were randomly divided into 3 groups: the normal group, the model group and the HKC group. The early DN model rats were induced by unilateral nephrectomy combined with intraperitoneal injection of streptozotocin, and administered with either HKC suspension or vehicle after modeling and for a period of 4 weeks. Changes in the incipient glomerular lesions-related parameters in urine and blood were analyzed. Kidneys were isolated for histomorphometry, immunohistochemistry, immunofluorescence and Western blotting (WB) at sacrifice. *In vitro*, murine mesangial cells (MCs) were used to investigate inhibitory actions of hyperoside (HYP), a bioactive component of HKC, on cellular hypertrophy-associated signaling pathway by WB, compared with rapamycin (RAP). For the early DN model rats, HKC ameliorated micro-urinary albumin, body weight and serum albumin, but had no significant effects on renal function and liver enzymes; HKC improved renal shape, kidney weight and kidney hypertrophy index; HKC attenuated glomerular hypertrophy, GBM thickening and mild mesangial expansion; HKC inhibited the phosphorylation of Akt, mTOR and p70S6K, and the protein over-expression of transforming growth factor-β1 in kidneys. *In vitro*, the phosphorylation of PI3K, Akt, mTOR and p70S6K in MCs induced by high-glucose was abrogated by treatment of HYP or RAP. On the whole, this study further demonstrated HKC safely and efficiently alleviates the early glomerular pathological changes of DN, likely by inhibiting Akt/mTOR/p70S6K signaling activity *in vivo* and *in vitro*, and provided the first evidence that HKC directly contributes to the prevention of the early DN.

## Introduction

The early diabetic nephropathy (DN) in both animal models and humans is characterized histologically by glomerular hypertrophy, glomerular basement membrane (GBM) thickening and mild mesangial expansion (Tervaert et al., 2010; Najafian et al., 2011). Since approximately 26 or 16.4% of the early DN patients in the United States (Afkarian et al., 2016) or in China (Liu, 2013) will progress to end-stage renal disease, the safe and effective therapeutic regiments for delaying the progression of DN are desired in clinic. However, so far, relatively little progress has been made in the treatment of the early DN patients (de Boer, 2017). Although angiotensin-converting enzyme inhibitors, angiotensin receptor blocker (Tuttle et al., 2014; Glassock, 2016) and some newly discovered drugs including glucagons-like peptide 1 agonists (Muskiet et al., 2017), dipeptidyl peptidase 4 inhibitors (Penno et al., 2016), sodium-glucose cotransporter 2 inhibitors (Zinman et al., 2015; Wanner et al., 2016) may help and treat the early DN patients by allowing tighter safer control of blood glucose level and by exerting beneficial direct effects on renal injuries, it is still unclear whether they can ameliorate the inchoate glomerular pathological changes including hypertrophic glomerulus, thickened GBM and mild mesangial expansion.

Mammalian target of rapamycin (mTOR) is a serine/threonine protein kinase, and its upstream signalings such as phosphatidylinositol-3-kinase (PI3K) and serine-threonine kinase (Akt) have been reported to regulate protein synthesis and cellular growth, specifically cellular hypertrophy (Yuan et al., 2011; Dibble and Cantley, 2015). PI3K/Akt/mTOR signaling pathway can directly regulate the functions of 70-kDa ribosomal protein S6 kinase (p70S6K) and eukaryotic initiation factor 4E-binding protein-1 (4EBP1), which are the important downstream regulators of ribosome protein synthesis, ribosome biogenesis, and mRNA translation initiation (Giasson and Meloche, 1995; Hall, 2016). Therefore, the activation of PI3K/Akt/mTOR signaling is thought to lead to an increase in the cellular capacity for protein synthesis and cellular hypertrophy. Sakaguchi et al. reported that rapamycin (RAP), a specific inhibitor of mTORC1 signaling, can attenuate renal enlargement and the enhanced phosphorylation of p70S6K in the kidneys of the early diabetic mice, suggesting the activation of PI3K/Akt/mTOR signaling and the phosphorylation level of p70S6K might play an important role in renal hypertrophy under hyperglycemia (Sakaguchi et al., 2006). Accordingly, targeting the activation of PI3K/Akt/mTOR signaling and the expression of phosphorylated p70S6K (p-p70S6K) in the kidneys could be therapeutic mechanisms for ameliorating the early glomerular lesions of DN.

Over the past 20 years, Huangkui capsule (HKC, the local name in China), a Chinese modern patent medicine extracted from *Abelmoschus manihot* (L.) medic (AM), has been approved by the China State Food and Drug Administration (Z19990040) for the conventional therapy of chronic glomerulonephritis (Guo et al., 2013; Zhang et al., 2014). HKC and its bioactive component hyperoside (HYP) can ameliorate proteinuria and renal dysfunction for patients and animal models with the early chronic kidney disease (CKD) (Chen et al., 2015; Ge et al., 2016). Recently, the increasing clinical evidences in China have been suggested that HKC at the safe and effective dose of 7.5 g/kg/day can reduce micro-urinary albumin (micro-UAlb) in the early DN patients and the IgA nephropathy patients (Liu et al., 2010; Li et al., 2017), and that its therapeutic action may be concerned with regulating transforming growth factor-β1 (TGF-β1) signaling *in vivo* (Tu et al., 2013), and inhibiting high-glucose (HG)-induced renal tubular epithelial-mesenchymal transition *in vitro* (Cai et al., 2017). In addition, our previous animal experiment as the first step revealed that, after the drug-intervention for 8 weeks, 2 g/kg/day dose of HKC can significantly attenuate the advanced renal fibrosis in the DN model rats induced by the unilateral nephrectomy combined with the intraperitoneal injection of streptozotocin (STZ) through regulating oxidative stress and p38 mitogen-activated protein kinase/Akt pathways (Mao et al., 2015). Despite these, up to present, there are still some important issues unresolved in the role of glomerular injuries in DN at the early stage treated by HKC, for instance, whether HKC can improve glomerular hypertrophy, GBM thickening and mild mesangial expansion by means of targeting PI3K/Akt/mTOR pathway and its signaling activity, and if yes, what are the underlying therapeutic mechanisms involved *in vivo* and *in vitro*.

Here, to address these issues, we designed the animal and cell experiments to verify these hypotheses that HKC at the dose of 2 g/kg/day may safely and efficiently alleviate the early glomerular pathological changes of the DN model rats, and inhibit the activation of PI3K/Akt/mTOR signaling pathway.

## Materials and methods

### HKC preparation and quality control

HKC purchased from Suzhong Pharmaceutical Group Co., Ltd. (Taizhou, China) is composed by the extracts from AM. One capsule of HKC contains 0.5 g of AM. The extracted method and productive process of HKC are both subjected to strict quality control, and the main components are subjected to standardization (Trendafilova et al., 2011; Xue et al., 2011). In addition, HKC is not only manufactured as granules after dynamic cycle extraction and concentration by evaporating and spray drying, but also monitored for the absence of contaminants (heavy metals, pesticides, hormone and mycotoxins) prior to the formulation. In this study, HKC (the batch number: 2014062703) was dissolved in distilled water (HKC suspension) and stored at 4°C before use.

**Figure 1:**
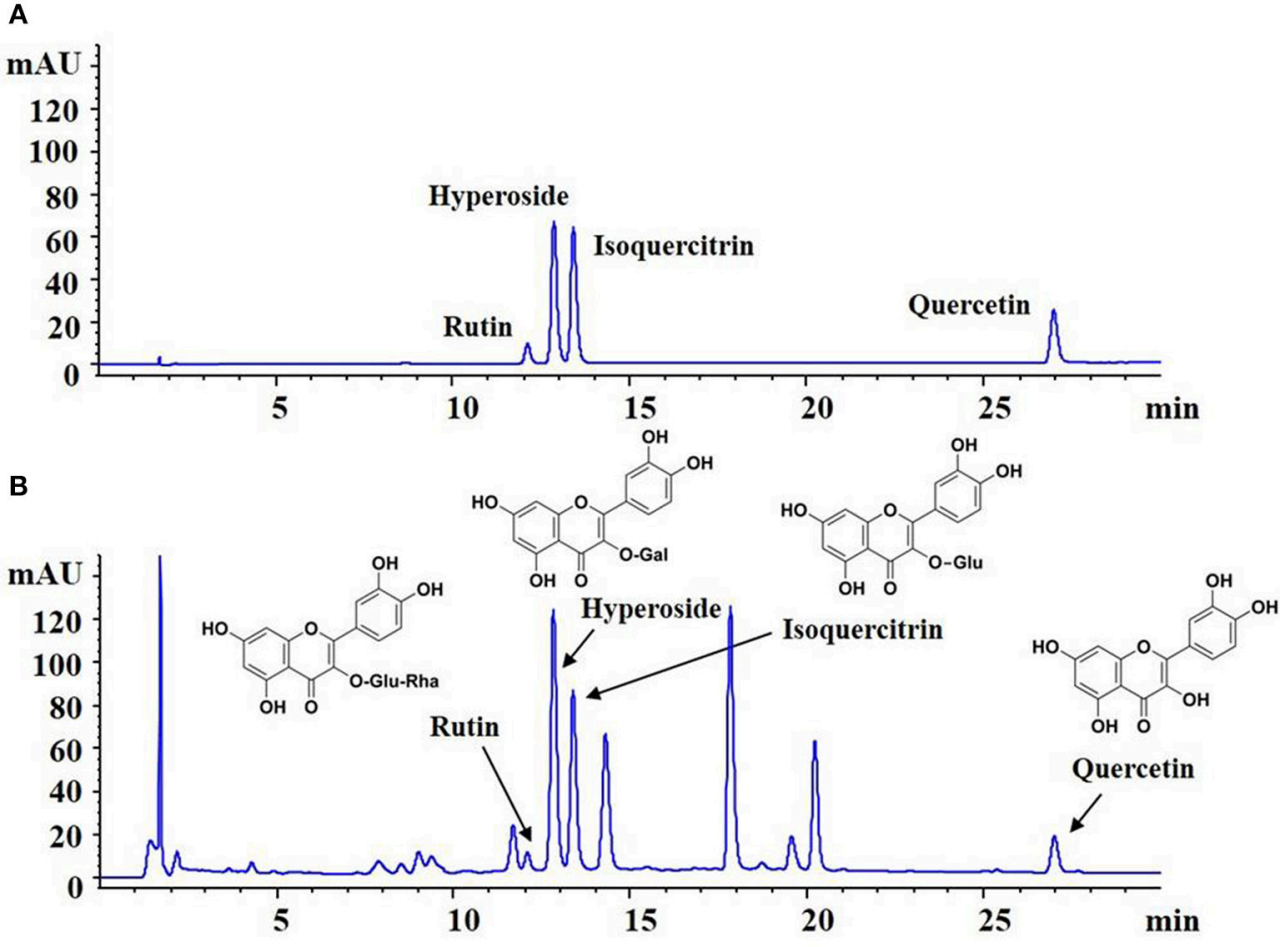
Fingerprint analysis of HKC by HPLC. **(A)** The chromatograms of mixed standards. **(B)** The samples of HKC.

The quality of HKC was examined with fingerprint analysis by high performance liquid chromatography (HPLC) as our previous study (Mao et al., 2015). As shown in Figure 1, the known bioactive components including flavonoids like rutin (C_27_H_30_O_16_; CAS: 153-18-4), hyperoside (C_21_H_20_O_12_; CAS: 482-36-0), isoquercitrin (C_21_H_20_O_12_; CAS: 482-35-9) and quercetin (C_15_H_10_O_7_; CAS: 117-39-5) (Figure 1A) in 5 batches exhibited high stability.

### Animals, drugs and reagents

All experiments were performed using the male Sprague-Dawley rats weighing from 200 to 220 g, purchased from Shanghai Jiesijie Experimental Animal Co., Ltd. [License No: SCXK (Shanghai) 2012-0006] and fed in the Experiment Animal Center of Nanjing Drum Tower Hospital, the Affiliated Hospital of Nanjing University Medical School. The surgical procedures and experimental protocol were approved by the Animal Ethics Committee of Nanjing University Medical School. HYP with the purity higher than 98% was purchased from Liangwei Biotechnology Co., Ltd. (Nanjing, China). STZ was purchased from Sigma-Aldrich (St. Louis, MO, USA). RAP and antibodies against Akt, phosphorylated Akt (p-Akt), p70S6K, p-p70S6K, mTOR, phosphorylated mTOR (p-mTOR), 4EBP1, phosphorylated 4EBP1 (p- 4EBP1), TGF-β1, Smad2, phosphorylated Smad2 (p-Smad2) and glyceraldehyde-3-phosphate dehydrogenase (GAPDH) were purchased from Cell Signaling Technology (Beverly, MA, USA). Antibody against nephrin was purchased from Abcam (Cambridge, UK). Antibodies against PI3K, phosphorylated PI3K (p-PI3K), proliferating cell nuclear antigen (PCNA) and horseradish peroxidase (HRP)-labeled IgG were purchased from Bioworld Technology (Louis Park, USA).

### Animal experimental protocols

**Figure 2:**
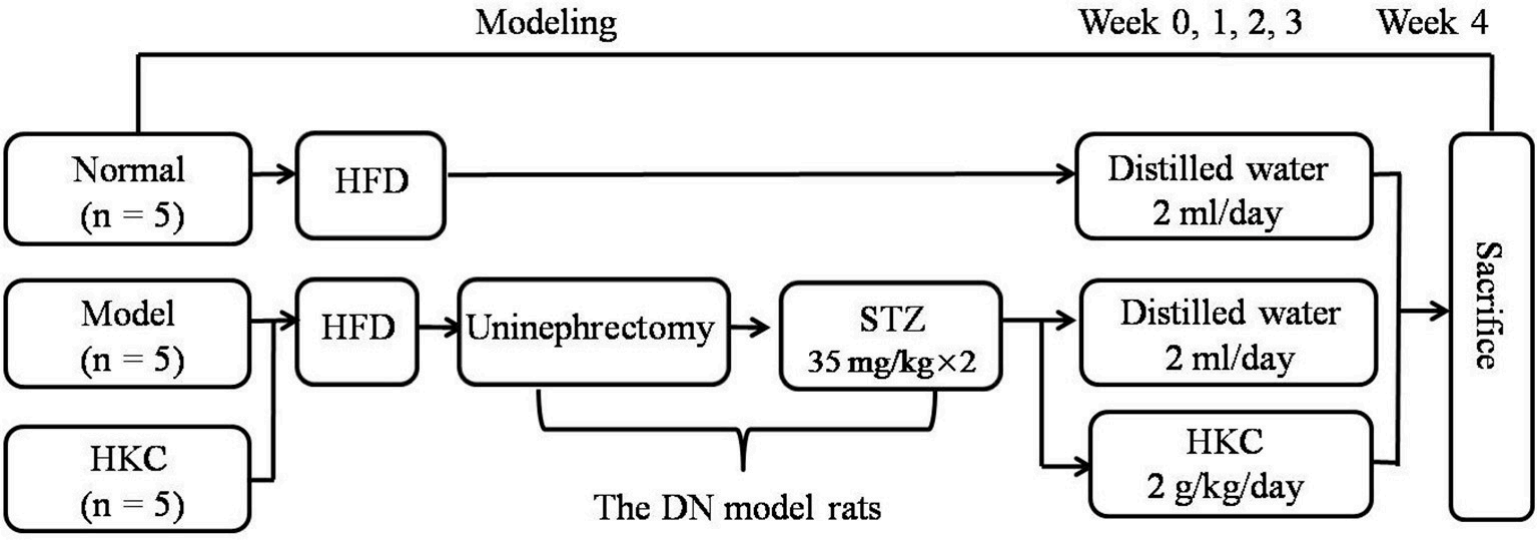
Animal experimental procedure.

The animal experiment procedure is illustrated in Figure 2. All rats were fed by the high-fat diet (HFD) purchased from Shanghai SLAC Laboratory Animal Co., Ltd (Shanghai, China) for 4 weeks. The DN rat models were established as described in our previous studies (Mao et al., 2015). Fifteen rats were divided into 3 groups, 5 rats in the normal group, 5 rats in the model group and 5 rats in the HKC group. In clinic, HKC at a dose of 7.5 g/day is used to treat a patient weighting 60 kg (Chen et al., 2012, 2016), which is equivalent to 1 g/kg/day in the rats. Given that the dose of 1 g/kg/day is set as the middle one, 2 g/kg/day is identified as the high dose. Following the second injection of STZ, HKC suspension was given to the rats in the HKC group by gastric gavage once a day for 4 weeks, while the rats in the model group and the normal group were treated with 2 ml vehicle (distilled water). Four weeks after administration, all rats were anesthetized and sacrificed through cardiac puncture. Blood samples and kidneys were collected for detection of various indicators.

### Rats' general status and biochemical parameters

Energy level, diet, water intake, fur color and activities of the rats in each group were observed daily. Body weight (BW), blood glucose (BG) and micro-UAlb of the rats were detected respectively before and every 1 or 2 weeks after modeling. The right kidneys of rats in each group were removed and weighed after cardiac puncture. Kidney hypertrophy index (KHI) was calculated according to the method described by Lane et al. (1990), that is KHI = kidney weight (KW)/BW. At the end of week 4 after the drug-intervention, the rats were anesthetized and blood samples (5 ml) were drawn from the heart. The biochemical parameters including serum albumin (Alb), serum creatinine (Scr), blood urea nitrogen (BUN), serum alanine transaminase (ALT) and serum aspartate transaminase (AST) were detected, respectively.

### Light microscopy examination

The tissue samples from renal cortex for light microscopy (LM) assessment were fixed with 10% neutral buffered formalin, embedded in paraffin, cut into 3-μm-thick sections and stained with the periodic acid-Schiff (PAS) or Masson reagent. Semiquantitative morphological studies of glomerular lesion were carried out by randomly selecting 20 full-sized glomeruli (80–100 μm) from each specimen. Glomerular cellular population (GCP) and glomerular volume (GV) were calculated with Image-Pro Plus 6.0 software (Media Cybernetic). Specifically, glomerular area was measured after glomerular capillary plexus profile was defined, and then GV was calculated in accordance with the method described by Lane et al. (1992), that is GV = area^1.5^ × 0.75 + 0.21. The results were confirmed by the pathological professional doctor.

### Electron microscopy investigation

The tissue samples from renal cortex for electron microscopy (EM) assessment were fixed in 2.5% glutaraldehyde in 0.1 mol/L phosphate buffer (PB) for several days at 4°C. After washing in PB and post-fixing in 1% OsO_4_ for 2 h, the fixed material was dehydrated through an ethanolpropylene oxide series and embedded in Araldite M. The ultrathin sections were prepared and stained with uranyl acetate and lead citrate, and then, investigated and photographed under a JEM-1011 transmission electron microscope (JEOL, Tokyo, Japan). Three glomeruli were selected randomly from each section. On the basis of the method described by Haas (2009), GBM thickness was directly measured and calculated with Image-Pro Plus 6.0 software (Media Cybernetic). The results were confirmed by the pathological professional doctor.

### Immunohistochemistry assay

Collagen type I (Col-I) and fibronectin (FN) were detected in 3 μm thick paraffin-embedded renal sections. For immunostaining of Col-I and FN antibodies against-Col-I and FN (Serotec, Oxford, UK) were used, respectively. Quantitative analysis of Col-I and FN was performed in a blinded fashion and expressed as cells/glomerular cross section. The results were confirmed by the pathological professional doctor.

### Immunofluorescence assay

The tissue samples from renal cortex for immunofluorescence (IF) studies were snap-frozen in precooled n-hexane and stored at −70°C. Frozen sections were cut into 3 μm thick with a cryostat and stained with an antibody against α-smooth muscle actin (α-SMA) (IgG2a) (Sigma, St. Louis, MO, USA). Fluoresceine isothiocyanate-conjugated antibody against mouse IgG2a (Southern Biotechnology Associates, Birmingham, AL, USA) was used as a secondary antibody (DACO A/S). The degree of α-SMA staining was scored from 0 to 4 + in 10 randomly selected glomeruli according to the method described by Raij et al. (1984). The results were confirmed by the pathological professional doctor.

### Western blotting analysis *in vivo*

Western blot (WB) analysis was performed as previously described (Mao et al., 2015). Renal tissues from the rats were isolated with phosphate-buffered saline including protease inhibitors (PI) and sequentially solubilized with 1% Triton X-100, RIPA buffer [0.1% sodium dodecyl sulfate (SDS), 1% sodium deoxycholate, 1% Triton X-100, 0.15 mol/L NaCl, and 0.01 mol/L ethylenediaminetetraacetic acid in 0.025 mol/L Tris-HCl, pH 7.2] with PI, and separated into Triton X-100-soluble (T), RIPA-soluble (R) and RIPA-insoluble (S) fractions. The RIPAI-insoluble fraction was solubilized with sodium dodecyl sulfate-polyacrylamide gel electrophoresis (SDS-PAGE) sample buffer (2% SDS, 10% glycerol and 5% 2-mercaproethanol in 0.0625 mol/L Tris-HCl, pH 6.8) (S fractions). Equal amounts of these sequentially solubilized fractions were subjected to SDS-PAGE with 7.5 or 10% acrylamide gel, and transferred onto a polyvinylidene fluoride membrane (Bio-Rad, Hercules, CA, USA) by electrophoretic trans-blotting for 30 min using Trans-Blot SD (Bio-Rad). After blocking with BSA, the strips of membrane were exposed to anti-PI3K, p-PI3K, Akt, p-Akt, mTOR, p-mTOR, p70S6K, p-p70S6K, 4EBP1, p-4EBP1, TGF-β1, Smad2, p-Smad2, nephrin, and GAPDH antibodies, respectively. They were washed and incubated with peroxidase-conjugated secondary antibodies for 1 h at room temperature. The bands were visualized by employing an alkaline phosphatase chromogen kit (5-bromo-4-chloro-3-indolil phosphate p-toluidine salt/nitro blue tetrazolium; Biomedica, AG, Staad, Switzerland). The density of the positive bands was quantitated by Densitograph (ATTO, Tokyo, Japan). The ratio of the densitometric signal of the molecules examined to that of GAPDH was determined. The data are shown as ratios relative to control findings and expressed as mean ± S.E. of 3 independent experiments.

### Mesangial cell culture and treatment

Murine mesangial cells (MCs) were kindly provided by Dr. Jian Yao (University of Yamanashi, Chuo, Japan) and cultured as described previously (Zhang et al., 2016). Briefly, MCs were cultured in RPMI-1640 medium supplemented with 10% heat-inactivated low endotoxin fetal bovine serum, penicillin/streptomycin and HEPES (GIBCO, California, USA) at 37°C in a humidified atmosphere containing 95% air and 5% CO_2_. MCs were treated with normal glucose (Normal, 5.5 mmol/L D-glucose) as control group, mannitol (MNT, 24.5 mmol/L MNT) as osmotic pressure control group, dimethylsulfoxide (DMSO, 0.1% DMSO) as DMSO control group and HG (30 mmol/L D-glucose) without or with HYP at the dose of 5 or 15 μg/ml and RAP at the dose of 20 nmol/L for 24, 48, or 72 h. Here, the low dose of HYP (5 μg/ml) and the high dose of HYP (15 μg/ml) were determined by the reference of Zhang et al. (2016). As HYP was dissolved in 0.1% DMSO, a similar volume of DMSO was added to DMSO control group.

### Cell viability assessment

MCs were cultured in 96-well microplates at a density of 5,000 cells/well. Twenty-four hours after cultivation, MCs were serum starved and treated with the different concentrations of HYP at 5, 10, 15, and 20 μg/ml or RAP at 10, 15, 20, and 25 nmol/L. After the exposure to drugs for 72 h, the cytotoxicity assay was performed using Cell Counting Kit-8 (CCK-8, Beyotime Institute of Biotechnology, Shanghai, China). The optical density (OD), the absorbance value at 450 nm, was read using a 96-well plate reader (BioTek, VT, USA), and the OD absorbance was proportional to the vitality of cells.

### Western blot analysis *in vitro*

MCs were treated in the different groups for 24, 48, or 72 h, respectively. After the treatment, cell lysates were separated by gel electrophoresis and blotted with antibodies against PI3K, p-PI3K, Akt, p-Akt, mTOR, p-mTOR, p70S6K, p-p70S6K, and GAPDH. The secondary antibody was HRP-conjugated anti-rabbit IgG antibody. WB analysis for cells was carried out according to our previous protocols (Tu et al., 2017).

### Statistics analysis

The differences among groups were analyzed by one-way analysis of variance (ANOVA), and LSD method was used for multiple comparison. Qualitative data were analyzed using Fisher's exact test as indicated. *P* < 0.05 was considered statistically significant.

## Results

### HKC ameliorates general condition and biochemical parameters of the early DN model rats

**Figure 3:**
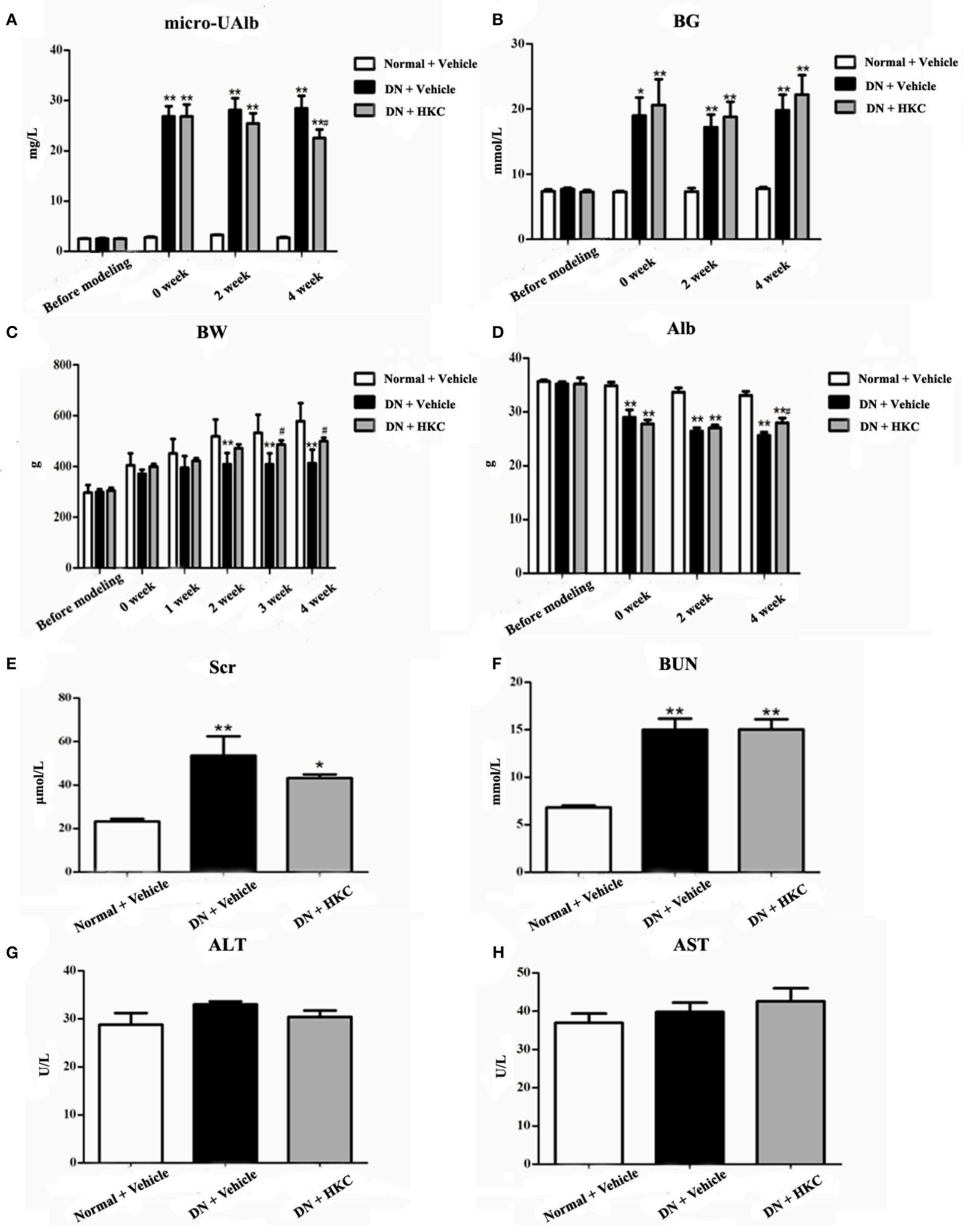
Effects of HKC on micro-UAlb **(A)**, BG **(B)**, BW **(C)**, Alb **(D)**, Scr **(E)**, BUN **(F)**, ALT **(G)**, and AST **(H)** of the early DN model rats. The data are expressed as mean ± S.E. ^*^P < 0.05, ^**^P < 0.01 vs. the normal group; ^#^*P* < 0.05 vs. the model group.

Throughout the experiment, the general status, micro-UAlb and BG of the normal group rats did not change significantly, while BW continued to rise. After modeling successfully, the rats both in the model group and the HKC group showed increased diet, water intake and urine volume, but low activity, dull fur and BW loss in different degrees. In which, the changes of the model group rats were particularly obvious. Compared with the normal group rats, BW of the model group rats rose slowly, but BG increased significantly and maintained above 16.7 mmol/L, moreover, micro-UAlb went up obviously (more than 20 mg/L), and the difference was statistically significant (*P* < 0.05). After HKC treatment for 4 weeks, micro-UAlb of the HKC group rats decreased, and compared with that of the model group rats, the difference was statistically significant (*P* < 0.05). At the end of 3 and 4 weeks after HKC treatment, BW of the HKC group rats increased, and compared with that of the model group rats, the difference was statistically significant (*P* < 0.05). However, HKC had no obvious effect on BG of the model group rats (Figures 3A,B).

Next, we investigated the effects of HKC on serum biochemical parameters in the 3 rat groups. As shown in Figure 3, Alb, Scr, BUN, ALT, AST of the normal group rats always kept at the normal levels during the whole experiment. After modeling successfully, Alb of the model group rats decreased gradually, while Scr and BUN increased slightly. At the end of 4 weeks after modeling, compared with those of the normal group rats, the differences were statistically significant (*P* < 0.01). After HKC treatment for 4 weeks, Alb of the HKC group rats increased significantly, and compared with that of the model group rats, the difference was statistically significant (*P* < 0.05). However, there were no obvious changes in Scr and BUN of the HKC group rats. In addition to these, the major liver enzymes including ALT and AST remained unchanged among the 3 rat groups (Figures 3D–H).

These results indicated that HKC could ameliorate micro-UAlb, BW and Alb of the early DN model rats, but had no significant effects on renal function and liver enzymes.

### HKC improves renal enlargement of the early DN model rats

**Figure 4:**
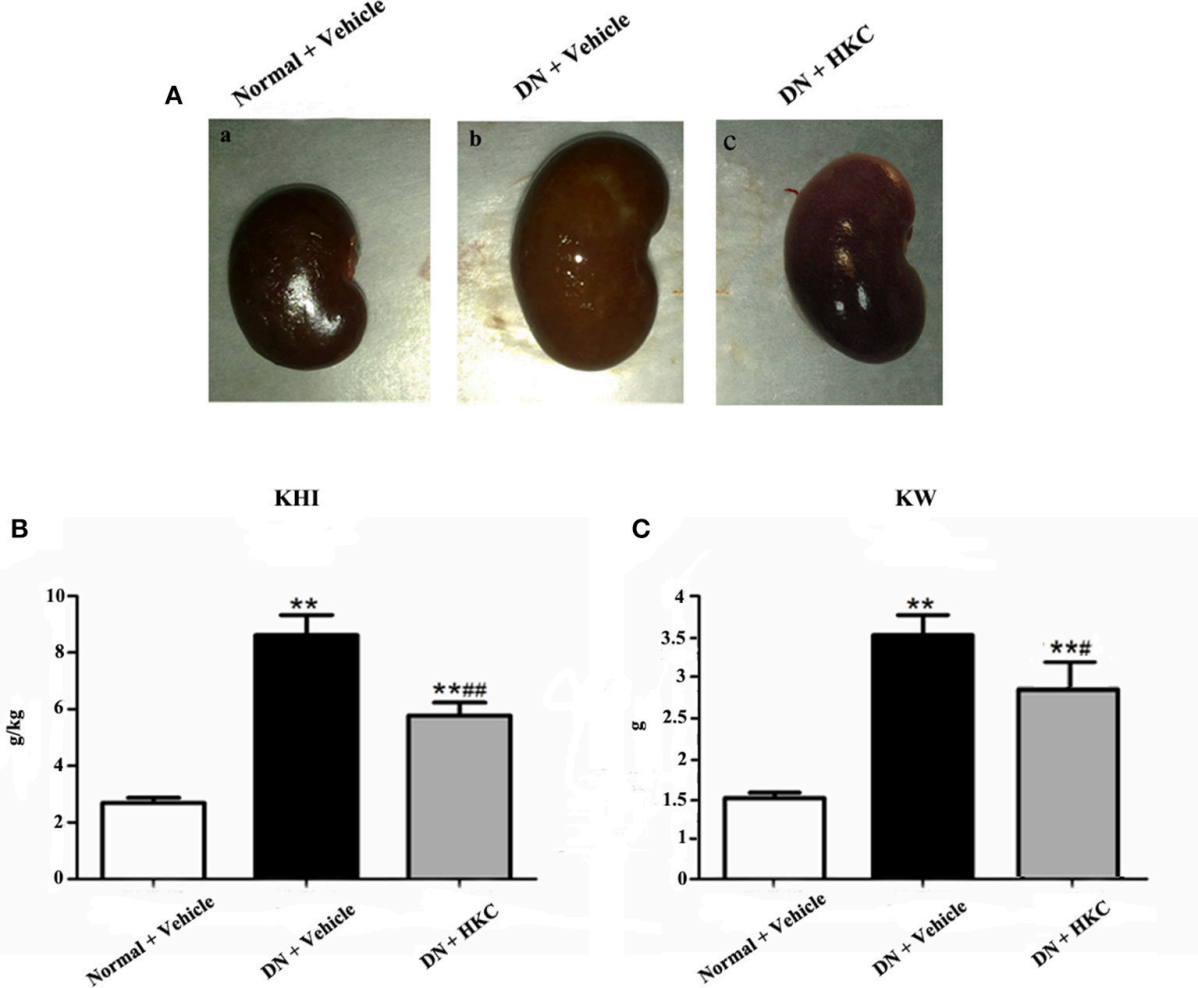
Effects of HKC on renal shape **(A)**, KHI **(B)**, and KW **(C)** of the early DN model rats. The data are expressed as mean ± S.E. ^**^P < 0.01 vs. the normal group; ^#^*P* < 0.05, ^##^*P* < 0.01 vs. the model group.

As shown in Figure 4, at the end of 4 weeks after modeling, renal shape, KHI and KW of the normal group rats were normal, while the kidneys of the model group rats significantly swelled and over-weighted. After HKC treatment for 4 weeks, renal shape KHI and KW of the HKC group rats were obviously improved, and compared with those of the model group rats, the differences were statistically significant (*P* < 0.05). Moreover, KHI decreased obviously, and compared with that of the model group rats, the difference was statistically significant (*P* < 0.01) (Figures 4A–C).

These results indicated that HKC could ameliorate renal shape, KW and KHI of the early DN model rats.

### HKC attenuates glomerular pathological changes of the early DN model rats

**Figure 5:**
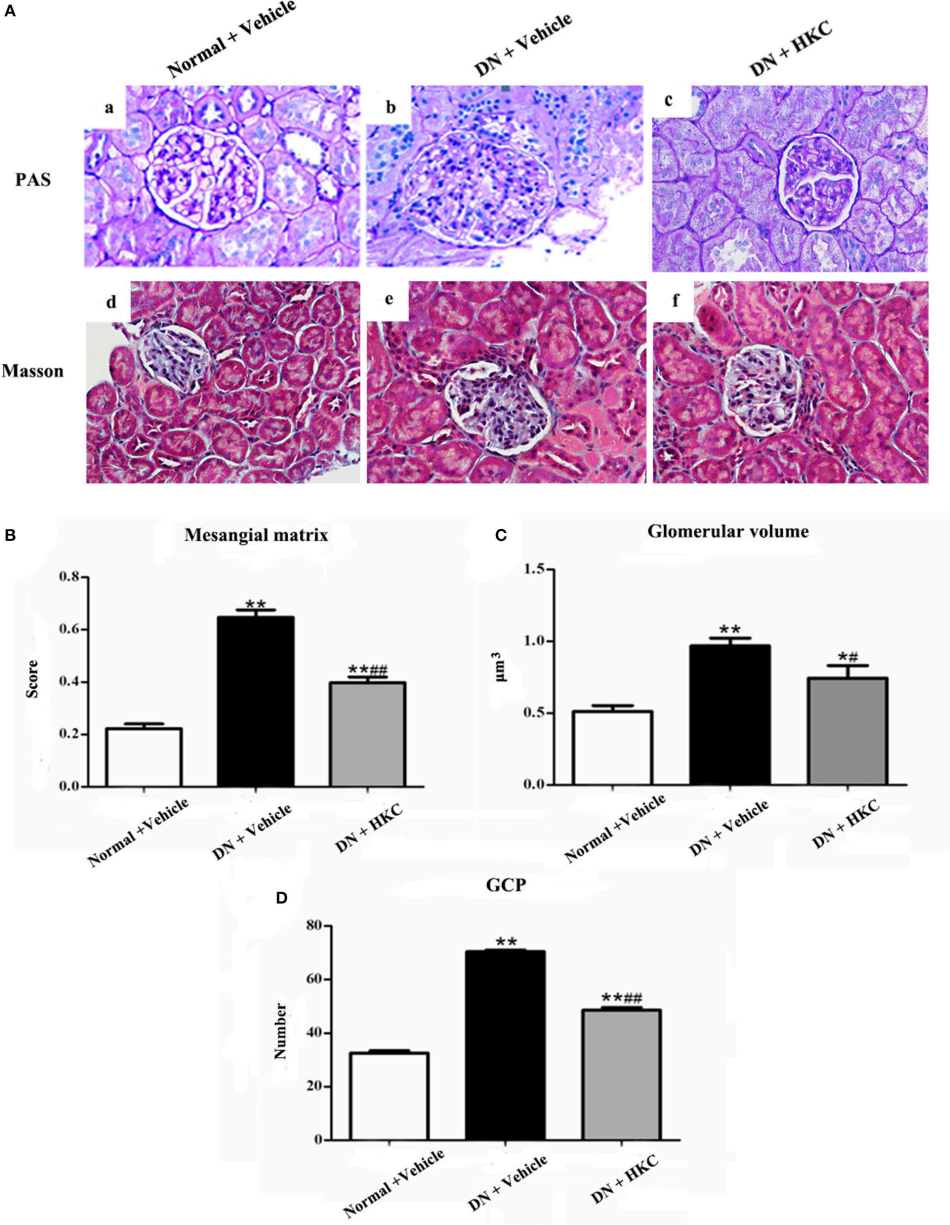
Effects of HKC on glomerular pathological changes of the early DN model rats. **(A)** Light microscopy; **(B)** Mesangial matrix score; **(C)** Glomerilar volume; **(D)** GCP; **(a–c)**: PAS staining × 400, **(d–f)**: Masson staining × 400. The data are expressed as mean ± S.E. ^*^P < 0.05, ^**^P < 0.01 vs. the normal group; ^#^*P* < 0.05, ^##^*P* < 0.01 vs. the model group.

**Figure 6:**
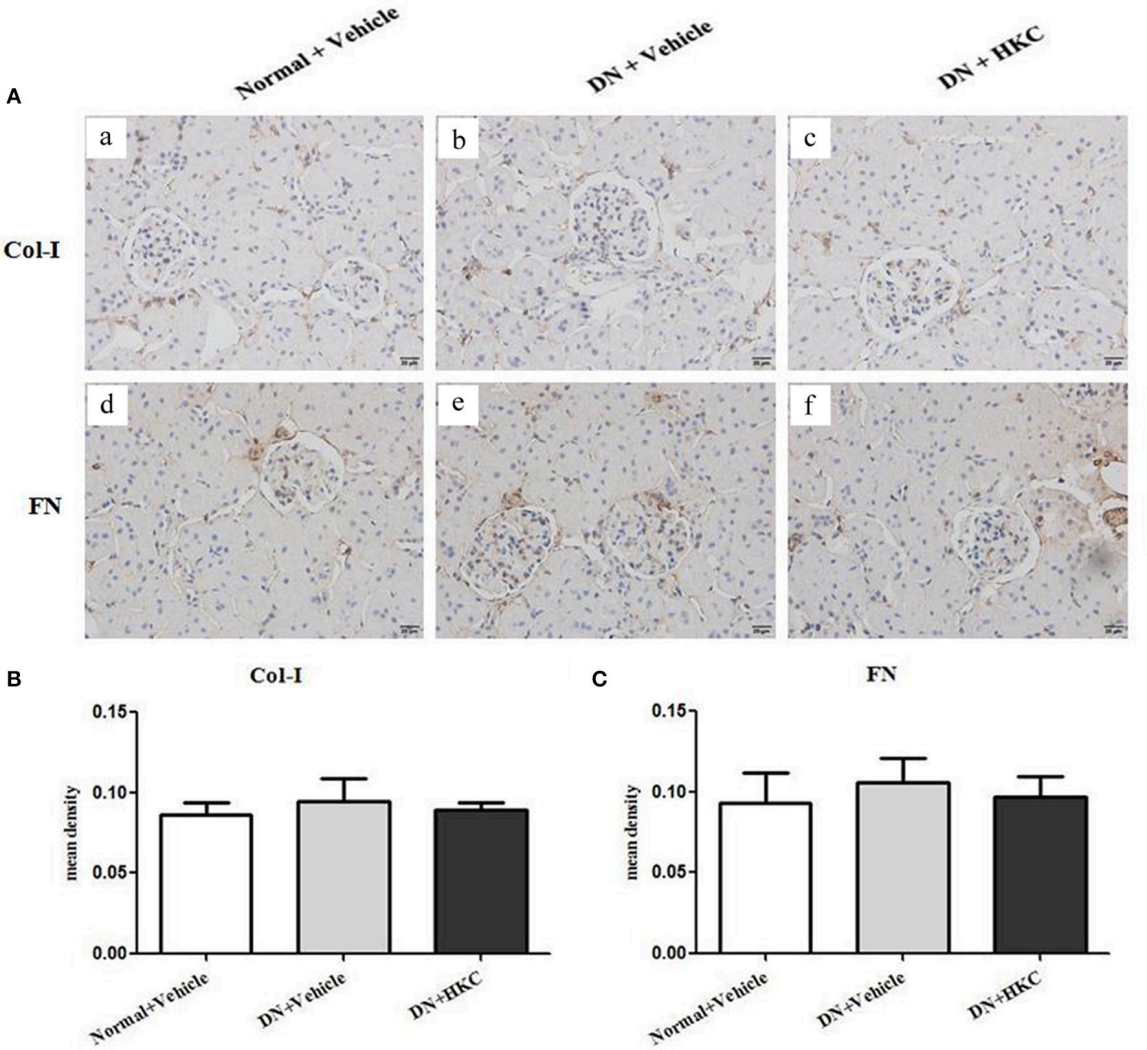
Effects of HKC on Col-I and FN immunohistochemical stainings in the kidneys of the early DN model rats. **(A)** Col-I **(a–c)** and FN **(d–f)** immunohistochemical staining in glomeruli (× 400); **(B)** Mean density of Col-I immunohistochemical staining; **(C)** Mean density of FN immunohistochemical staining. The data are expressed as mean ± S.E.

Firstly, we investigated the effects of HKC on glomerular form, glomerular volume, mesangial matrix and GCP in the 3 rat groups. As shown in Figure 5, at the end of 4 weeks after modeling, upon the observation under LM, the entire and clear glomerular structure and open glomerular capillary loops were seen in the normal group rats. By contrast, hypertrophic glomerular form, increased glomerular volume and GCP and mild mesangial matrix expansion of the model group rats were found respectively, and the differences were statistically significant (*P* < 0.01). After HKC treatment for 4 weeks, the early glomerular pathological changes of the HKC group rats were improved significantly, and compared with those of the model group rats, the differences were statistically significant (*P* < 0.01 or *P* < 0.05) (Figures 5A–D). Here, notably, neither Masson-staining of collagen nor immunostainings of Col-I and FN (Figures 6A–C) was detected obviously in glomeruli of the 3 rat groups.

**Figure 7:**
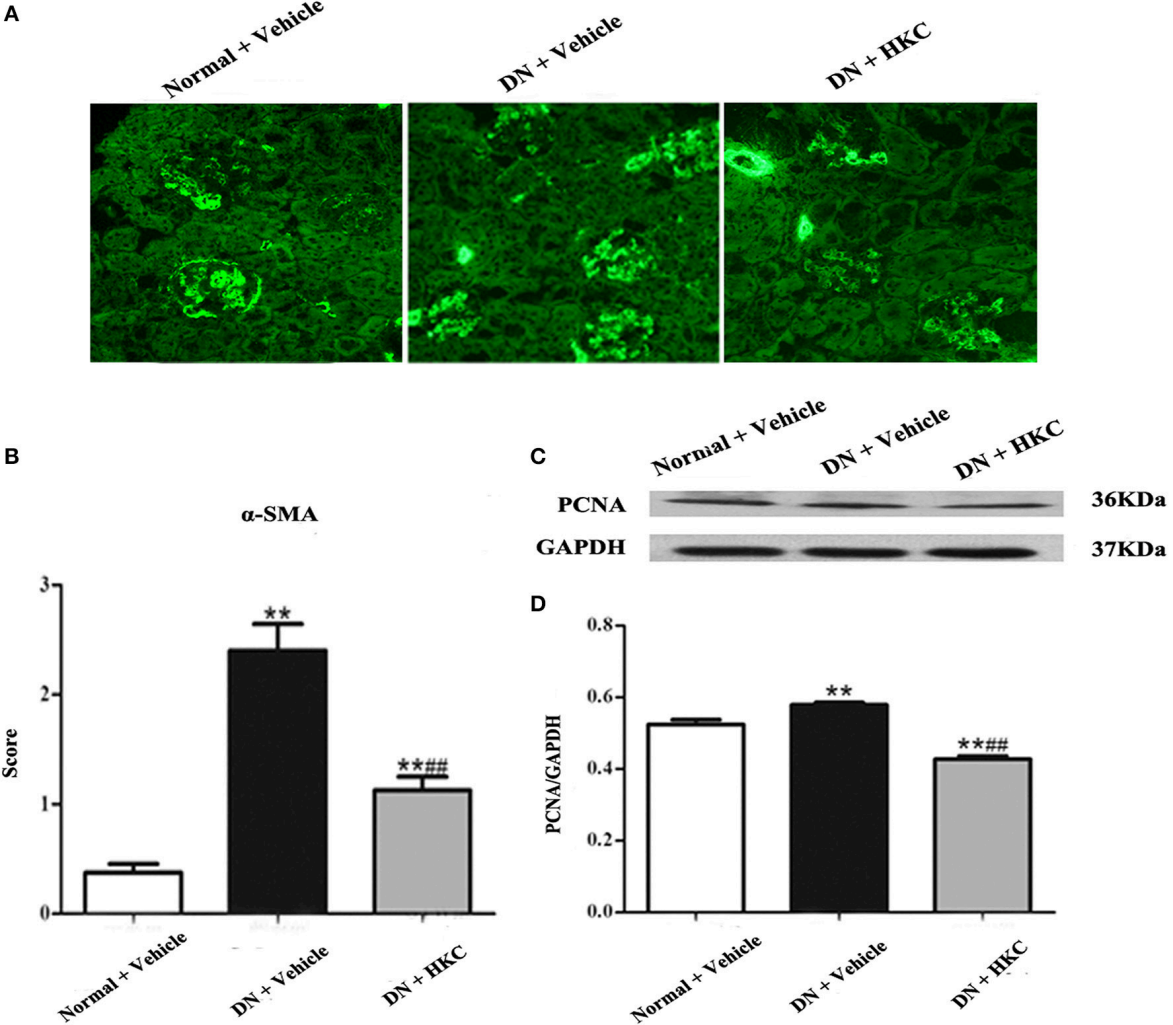
Effects of HKC on α-SMA fluorescence staining in glomeruli and PCNA protein expression in the kidneys of the early DN model rats. **(A,B)** α-SMA fluorescence staining in glomeruli (× 400) and its score, **(C,D)** WB analysis (upon) for PCNA protein expression in the kidney, with the quantification (below). The data are expressed as mean ± S.E. ^**^P < 0.01 vs. the normal group; ^##^*P* < 0.01 vs. the model group.

Then, we observed the effects of HKC on the fluorescence staining of α-SMA in glomeruli and the protein expression of PCNA in the kidneys of the 3 rat groups. PCNA and α-SMA are the acknowledged markers of mesangial cell proliferation. At the end of 4 weeks after modeling, the normal group rats had weak expressions of α-SMA in glomeruli and PCNA in the kidneys. By contrast, the extent of α-SMA fluorescence staining in glomerulai and the level of PCNA protein expression in the kidneys of the model group rats increased significantly, and the differences were statistically significant (*P* < 0.01). After HKC treatment for 4 weeks, the expressions of α-SMA in glomeruli and PCNA in the kidneys of the HKC group rats were decreased significantly, and compared with those of the model group rats, the differences were statistically significant (*P* < 0.01) (Figure 7).

**Figure 8:**
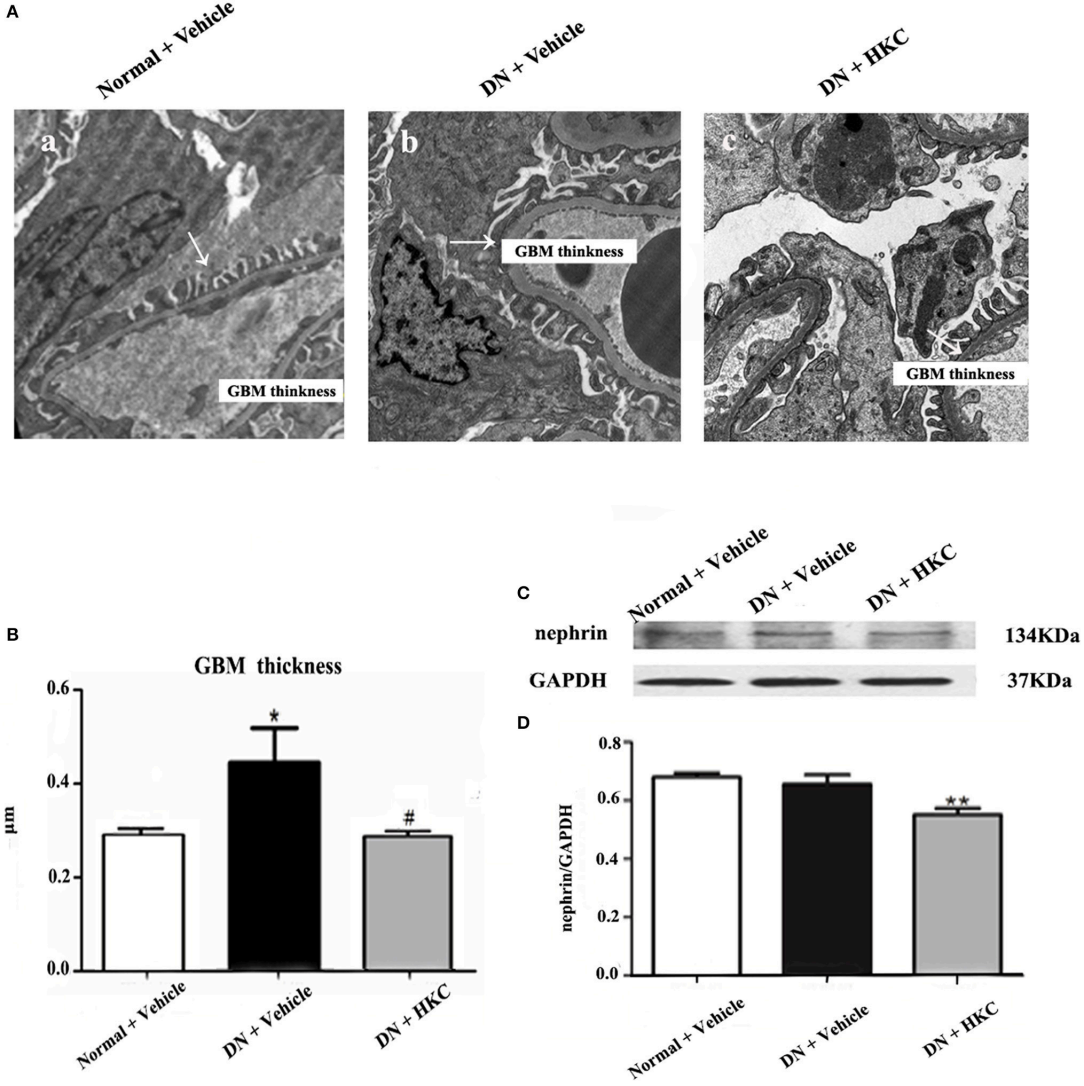
Effects of HKC on GBM thickness, foot process form and nephrin protein expression in the kidneys of the early DN model rats. **(A)** Foot process ultrastructure (× 1,700); **(B)** GBM thickness; **(C,D)** WB analysis (upon) for nephrin protein expression in the kidney, with the quantification (below). The data are expressed as mean ± S.E. ^*^P < 0.05, ^**^P < 0.01 vs. the normal group; ^#^*P* < 0.05 vs. the model group.

Thirdly, we examined the effects of HKC on GBM thickness, foot process form and nephrin protein expression in the kidneys of the 3 rat groups. As shown in Figure 8, at the end of 4 weeks after modeling, the thickness of GBM, the form of foot process and the protein expression level of nephrin in the kidneys of the normal group rats did not change. By contrast, GBM thickening and foot process loss and effacement were detected in the model group rats, thereinto, GBM thickening is obvious, and compared with that of the normal group rats, the difference was statistically significant (*P* < 0.05). Here, it is noted that the protein expression of nephrin in the kidneys of the 3 rat groups remained unchanged. After HKC treatment for 4 weeks, GBM thickening of the HKC group rats decreased, and compared with that of the model group rats, the difference was statistically significant (*P* < 0.05). However, unfortunately, the significant improvement in foot process loss and effacement and nephrin protein expression in the kidneys of the HKC group rats was not found.

These results indicated that HKC could ameliorate glomerular pathological changes of the early DN model rats, such as glomerular hypertrophy, GBM thickening and mild mesangial expansion.

### HKC inhibits activation of mTOR signaling by PI3K/Akt pathway, not by TGF-β1/Smad2 pathway in the kidneys of the early DN model rats

**Figure 9:**
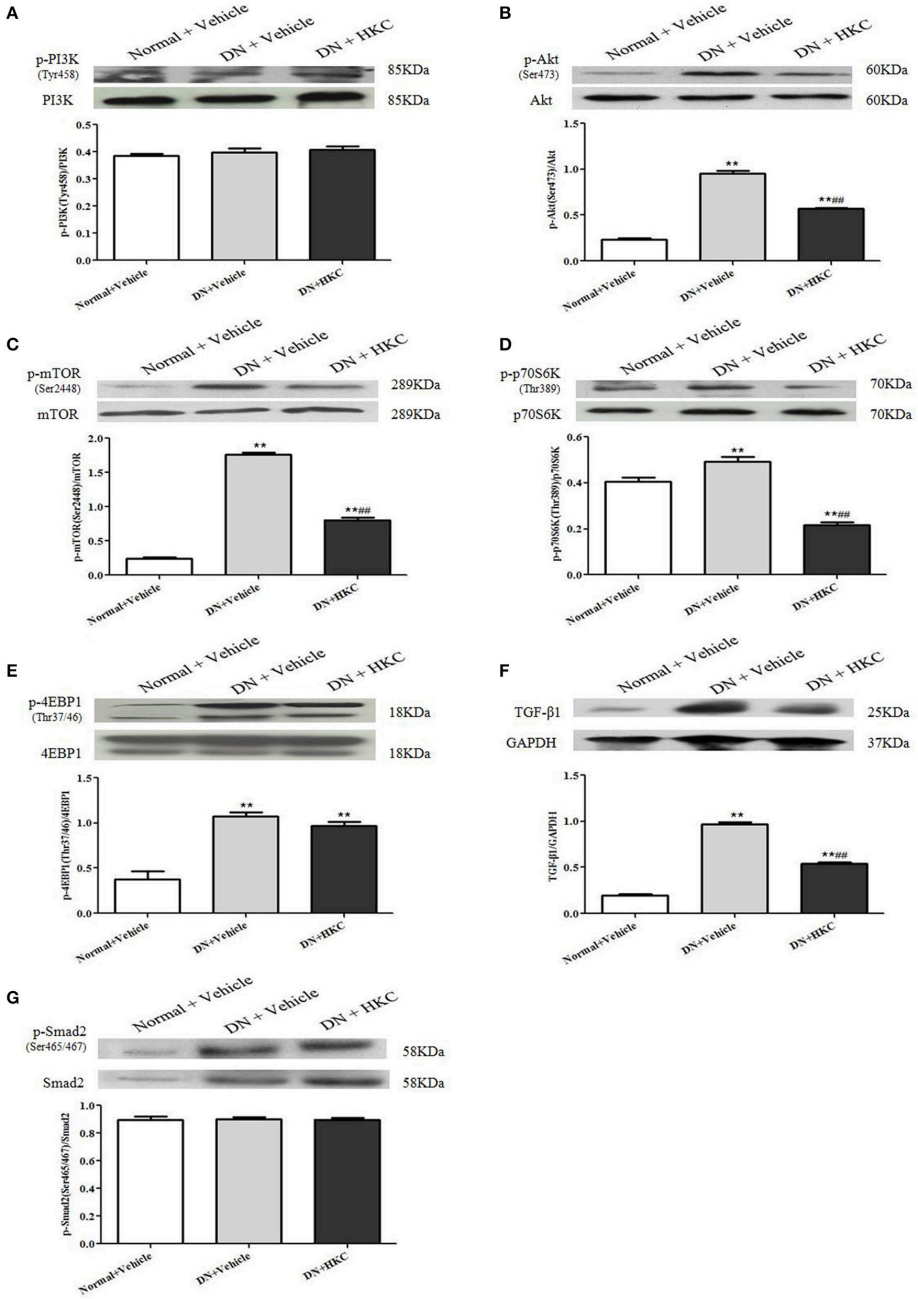
Effects of HKC on the key signaling molecules of PI3K/Akt/mTOR and TGF-β1/Smad2 signaling pathways in the kidneys of the early DN model rats. **(A–E)** WB analysis (upon) for the protein expressions of PI3K, p-PI3K, Akt, p-Akt, mTOR, p-mTOR, p70S6K, p-p70S6K, 4EBP1, and p-4EBP1, with the quantification (below); **(F,G)** WB analysis (upon) for the protein expressions of TGF-β1, Smad, p-Smad2 and GAPDH, with the quantification (below). The data are expressed as mean ± S.E. ^**^P < 0.01 vs. the normal group; ^##^*P* < 0.01 vs. the model group.

PI3K/Akt/mTOR and TGF-β1/Smad2 signaling pathways play the different roles in the progression of DN. The key signaling molecules of PI3K/Akt/mTOR and TGF-β1/Smad2 pathways include p-PI3K (Tyr458), p-Akt (Ser473), p-mTOR (Ser2448), p-p70S6K (Thr389), p-4EBP1 (Thr37/46), TGF-β1 and p-Smad2 (Ser465/467). At the end of 4 weeks after modeling, there was no significant change in p-PI3K protein expression level among the 3 rat groups (Figure 9A), but the protein expression levels of p-Akt, p-mTOR, p-p70S6K, p-4EBP1, TGF-β1 and p-Smad2 in the kidneys of the model group rats were up-regulated significantly, and compared with those of the normal group rats, the differences were statistically significant (*P* < 0.01) (Figures 9B–G). After HKC treatment for 4 weeks, the protein expression levels of p-Akt, p-mTOR, p-p70S6K, and TGF-β1 in the kidneys of the HKC group rats were down-regulated significantly, and compared with those of the model group rats, the differences were statistically significant (*P* < 0.01) (Figures 9B–D). Despite this, the protein expressions of p-Smad2 and p-4EBP1 in the kidneys of the HKC group rats and the model group rats remained unchanged within 4 weeks after vehicle or drug-intervention (Figures 9E,G).

These results indicated that HKC could inhibit the protein over-expressions of p-Akt, p-mTOR, p-p70S6K, and TGF-β1 in the kidneys of the early DN model rats, but had no significant effect on the protein expressions of p-PI3K, p-Smad2, and p-4EBP1.

### Hyperoside abrogates phosphorylation of PI3K, Akt, mTOR and P70S6K induced by high-glucose in the cultured mesangial cells *in vitro*

**Figure 10:**
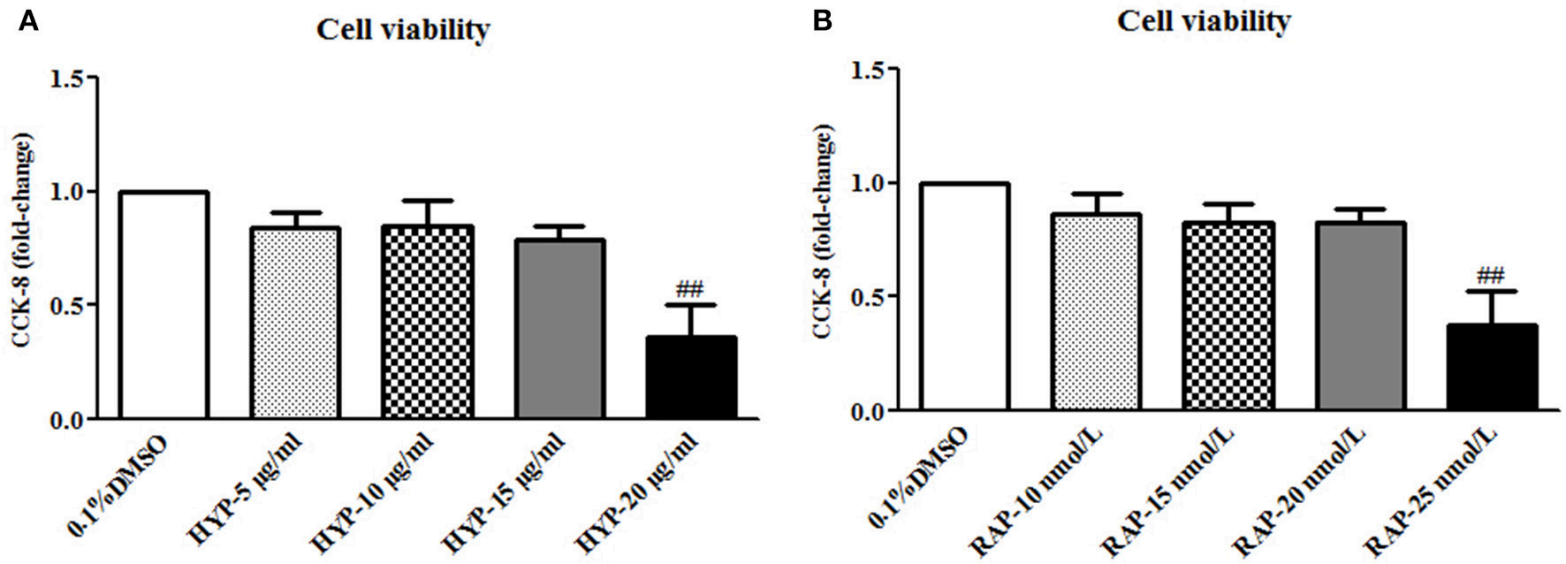
The cell viability in the cultured mesangial cells. **(A)** The MCs were exposed to 0.1% DMSO and HYP at 5, 10, 15, and 20 μg/ml for 72 h; **(B)** The MCs were exposed to 0.1% DMSO and RAP at 10, 15, 20, and 25 nmol/L for 72 h. The data are expressed as mean ± S.E. ^##^*P* < 0.01 vs. the treatment of HYP at 15 μg/ml or RAP at 20 nmol/L.

**Figure 11:**
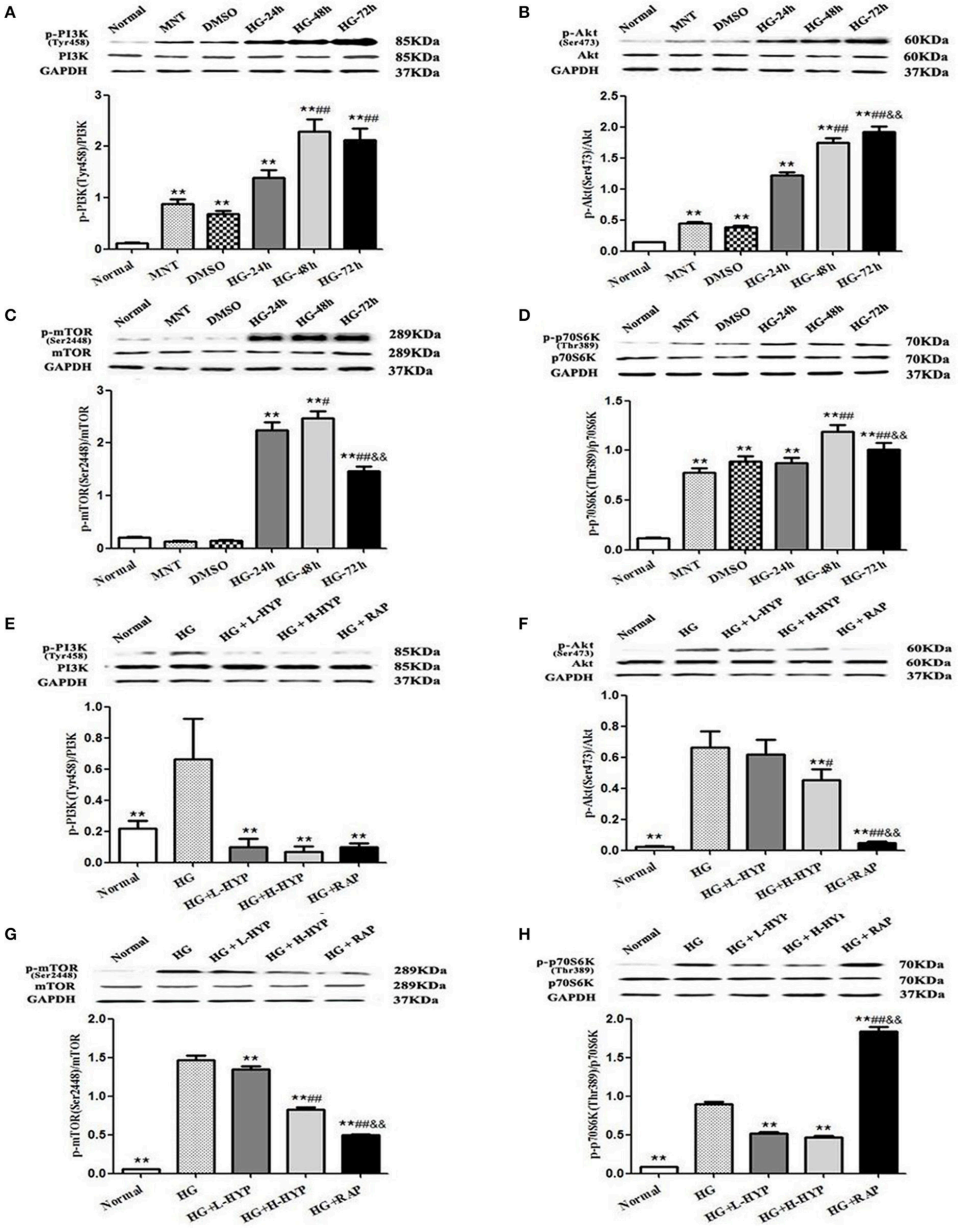
Effects of HYP on the phosphorylation of PI3K, Akt, mTOR, and p70S6K induced by HG in the cultured mesangial cells *in vitro*. **(A–D)** WB analysis (upon) for the protein expressions of PI3K, p-PI3K, Akt, p-Akt, mTOR, p-mTOR, p70S6K, and p-p70S6K treated with normal glucose (Normal), MNT, DMSO and HG at 24, 48, and 72 h, respectively, with the quantification (below); **(E–H)** WB analysis (upon) for the protein expressions of PI3K, p-PI3K, Akt, p-Akt, mTOR, p-mTOR, p70S6K, and p-p70S6K after the treatment with normal glucose, HG, L-HYP, H-HYP, and RAP at 72 h, respectively, with the quantification (below). The data are expressed as mean ± S.E. **(A–D)** ^**^P < 0.01 vs. the normal glucose (Normal) group; ^#^*P* < 0.05, ^##^*P* < 0.01 vs. the HG-24 h group; ^&&^*P* < 0.01 vs. the HG-48 h group. **(E–H)** ^**^P < 0.01 vs. the HG group; ^#^*P* < 0.05, ^##^*P* < 0.01 vs. the HG+L-HYP group; ^&&^*P* < 0.01 vs. the HG+H-HYP group.

Prior to the formal cellar experiments, the cytotoxicity of HYP and RAP on the cultured MCs was analyzed using CCK-8. As shown in Figure 10, the cell viabilities were significantly decreased under the highest concentrations of HYP at 20 μg/ml (Figure 10A) and RAP at 25 nmol/L (Figure 10B) compared with the 15 μg/ml dose of HYP and the 20 nmol/L dose of RAP, respectively. According to these results, the suitable doses of HYP (5 and 15 μg/ml) and RAP (20 nmol/L) were selected respectively. Coincidentally, these drug concentrations were similar to the report of Zhang et al. (2016). To confirm further whether HG affects the phosphorylation of PI3K, Akt, mTOR, and p70S6K *in vitro*, we tested the protein expressions of PI3K, p-PI3K, Akt, p-Akt, mTOR, p-mTOR, p70S6K, and p-p70S6K in the cultured MCs treated with HG at 24, 48, and 72 h, compared with the treatment of MNT or DMSO. The results showed that HG increased the protein expressions of p-PI3K, p-Akt, p-mTOR, and p-p70S6K in the cultured MCs in a time-dependent manner, suggesting HG could induce the phosphorylation of PI3K, Akt, mTOR, and p70S6K *in vitro* (Figures 11A–D). In addition, it is noted that the treatment with HYP at the different doses and RAP (mTORC1 inhibitor) at 72 h significantly down-regulated HG-induced changes in the protein expressions of p-PI3K, p-Akt, p-mTOR, and p-p70S6K in the cultured MCs, compared with the treatment of HG (Figures 11E–H). In which, the suppressive effect of H-HYP (15 μg/ml) on the phosphorylation of p70S6K was better than RAP, and the difference was statistically significant (*P* < 0.01). Whereas, the repressive actions of RAP on the phosphorylation of Akt and mTOR were better than H-HYP, and the differences were statistically significant (*P* < 0.01).

These results indicated that HYP, different from RAP, could inhibit the phosphorylation of PI3K, Akt, mTOR, and p70S6K induced by HG in the cultured mesangial cells *in vitro*.

## Discussion

In the present study, using a modified DN rat model and the murine MCs, we emphatically demonstrated that HKC at the safe and effective dose of 2 g/kg/day can not only improve micro-UAlb and renal enlargement but also alleviate the early glomerular pathological changes including glomerular hypertrophy, GBM thickening and mild mesangial expansion, and that, more importantly, these ameliorative effects are closely related with the inhibition of Akt/mTOR/p70S6K signaling activity *in vivo* and *in vitro*.

A well-defined sequence of glomerular injuries in the early DN has been identified (Tervaert et al., 2010). The histologically characteristics of DN in both animal models and humans are 3 lesions, namely hypertrophic glomerulus, thickened GBM and mild mesangial expansion. In which, glomeruli may show only hypertrophy or be of normal size without any lesions in the earliest stage. GBM thickening is due to the increased accumulation of mesangial expansion, and progresses with the increased duration of diabetes (Najafian et al., 2011). For these reasons, in this study, we firstly tried to establish the useful and early DN rat model by unilateral nephrectomy combined with STZ intraperitoneal injections with the low doses of 35 mg/kg BW for twice at 72 h-interval. Our results showed that, these DN model rats were well-replicated hyperglycemia (more than 16.7 mmol/L), micro-UAlb (more than 20 mg/L), renal enlargement and the early glomerular pathological changes such as hypertrophic glomerulus, thickened GBM and mild mesangial expansion within 4 weeks after modeling. Furthermore, it is noteworthy that most of the DN model rats did not exhibit a mass of proteinuria, renal dysfunction and the typical glomerular sclerosis marked by the severe collagen accumulation and the significant stainings of FN and Col-I in glomeruli, which are the recognized glomerular damaged features in the middle and advanced stages of DN. Whereupon, we considered that this modified DN rat model should be helpful in unraveling the early glomerular injuries and finding the novel therapeutic drugs in human DN.

In our previous studies, we described that HKC, a Chinese modern patent medicine in the long-time clinical practice (Zhang et al., 2014; Chen et al., 2015, 2016), with the dose of 2 g/kg/day is renoprotective via attenuating the advanced renal fibrosis after the drug administration for 8 weeks in the DN rat model (Mao et al., 2015). By comparison, in the present study, on the basis of the inchoate glomerular damaged animal model induced by unilateral nephrectomy and STZ injections, we further expounded whether HKC could improve the early glomerular pathological changes within 4 weeks after modeling. Our data indicated that HKC significantly ameliorated renal shape, KW and KHI, and improved hypertrophic glomerular form, increased glomerular volume and GCP and mild mesangial matrix expansion of the early DN model rats. In addition to these, HKC decreased the fluorescence staining of α-SMA in glomeruli and the protein expression of PCNA in the kidneys, which are the acknowledged markers of mesangial cell proliferation leading to glomerular hypertrophy in the early stage of DN (Hall et al., 1990; Cheng et al., 2014). Thus, we confirmed that HKC at the dose of 2 g/kg/day could attenuate glomerular hypertrophy *in vivo*, which is one of the main glomerular pathological characteristics of the early DN.

To our knowledge, GBM thickening is another characteristic pathological change in type 1 and type 2 DN and increases with the duration of the disease (White and Bilous, 2000). GBM thickening is a consequence of mesangial matrix expansion, with the increased deposition of normal mesangial matrix components such as Col-I and FN. Such accumulations result from the increased production of these proteins, their decreased degradation, or a combination of the two (Kim et al., 1991). In this study, we found that GBM thickening and mild mesangial expansion, as well as the ultrastructural changes of podocyte such as foot process loss and effacement were detected respectively in the early DN model rats, and ameliorated distinctly after HKC treatment for 4 weeks, at the same time, micro-UAlb of the DN model rats also decreased in a different degree. Interestingly, similar to our finding, An et al. (2017) reported recently that the pre-treatment of HYP, a bioactive component of HKC, prevents GBM damage in DN by inhibiting podocyte heparanase expression. Therefore, we had reasons to believe that HKC at the dose of 2 g/kg/day and its bioactive component could alleviate glomerular pathological changes of the early DN model rats.

Animal and clinical studies have reported the roles for PI3K/Akt/mTOR and TGF-β1/Smad2 signaling pathways in the early stage of DN, and the blockade of these pathways slows the progression and development of DN (Gangadharan-Komala et al., 2016; Li et al., 2016). Sakaguchi et al. (2006) reported that mTOR signaling is activated in the early DN model mice and the cultured mouse proximal tubule cells, and that mTOR signaling causes renal enlargement and glomerular hypertrophy through regulating the phosphorylation of p70S6K. Nagai et al. (2005) also reported that the activated PI3K/Akt/mTOR signaling and the enhanced p-p70S6K expression are detected in glomeruli in diabetes-induced by STZ injection. In addition, Lu et al. (2015) reported that the extract of *Ginkgo biloba* leaves (GbE) prevents renal fibrosis in rats with DN-induced by STZ injection, which is most likely to be associated with its abilities to inhibit Akt/mTOR signaling pathway. Hence, we proposed that regulating the activation of PI3K/Akt/mTOR and/or TGF-β1/Smad2 pathways in this DN rat model is a successful way to identify the therapeutic mechanisms *in vivo* of HKC on attenuating the early glomerular pathological changes. Our data clearly indicated that the increased protein expressions of p-Akt (Ser473), p-mTOR (Ser2448), p-p70S6K (Thr389), p-4EBP1 (Thr37/46), TGF-β1 and p-Smad2 in the kidneys were obviously revealed in the DN model rats, in the meantime, concomitant with the appearance of glomerular pathological changes including glomerular hypertrophy, GBM thickening and mild mesangial expansion. These results forcefully suggested that Akt/mTOR and TGF-β1/Smad2 signaling pathways are activated in this DN rat model, and there is a strong causality *in vivo* between the key signaling molecular expressions in these signaling pathways and the early glomerular injuries. More importantly, we also found that HKC simultaneously inhibited the activation of Akt/mTOR pathway as well as the protein expression of p-p70S6K in the kidneys of the DN model rats within 4 weeks. By contrast, the protein expressions of p-Smad2 as a key signaling molecule of TGF-β1/Smad2 pathway and p-4EBP1 as a downstream target of mTOR in the kidneys remained unchanged after HKC treatment. Here, without using the mTOR inhibitor, it is puzzling why the activation of 4EBP1 was not affected after the treatment with HKC. On the whole, using an intravital DN rat model, we suggested that HKC *in vivo* at the dose of 2 g/kg/day could only inhibit the activation of Akt/mTOR signaling and the phosphorylation level of p70S6K in the kidneys. Interestingly, consistent with the *in vivo* results basically, based on the murine MCs, we preliminarily confirmed the given doses of HYP, a bioactive component of HKC, could also inhibit the activation of PI3K/Akt/mTOR/p70S6K signaling axis induced by HG *in vitro*, which is a little bit different from RAP (mTORC1 inhibitor). Recently NOD-like receptor family CARD domain containing 3 (NLRC3) has been identified as the upstream negative molecule in PI3K/Akt/mTOR signaling axis to inhibit the activation of PI3K, Akt and mTOR in cancer (Karki et al., 2016). If so in the kidneys under the HG status, we boldly hypothesize HKC or HYP at the upstream can inhibit NLRC3 to regulate PI3K/Akt/mTOR signaling axis. Further detailed analyses *in vitro* and *in vivo* of NLRC3 are needed to address this hypothesis.

Finally, we need to bring up 2 additional points. First, HKC, a natural anti-nephritic phytomedicine, did not lower hyperglycemia in this DN rat model. We unavoidably thought of the cause-and-effect of relationship between hyperglycemia and the early glomerular pathological changes. Some studies have showed that GBM thickening and glomerular hypertrophy are described as a “pre-diabetic” lesion (Mac-Moune-Lai et al., 2004). We thereby believed that HKC has the renoprotective action, completely independent of lowering hyperglycemia. Second, 2 g/kg/day dose of HKC has been proved effective in attenuating the advanced renal fibrosis in the DN model rats (Mao et al., 2015). To exclude the side effects of HKC at such high dose on hepatic damage in this DN rat model, we emphatically compared the levels of serum ALT and AST in 3 group rats. Our results revealed that serum ALT and AST in each group had no obvious changes, suggesting HKC at the dose of 2 g/kg/day has no negative effect on liver function *in vivo*.

In conclusion, the results of this report further demonstrated that HKC at the safe and effective dose of 2 g/kg/day can alleviate the early glomerular pathological changes of the DN model rats including glomerular hypertrophy, GBM thickening and mild mesangial expansion, likely by the inhibition of Akt/mTOR/p70S6K signaling activity *in vivo* and *in vitro*. This study provided the first evidence that HKC directly contributes to the prevention of the early DN.

## Author contributions

Y-GW and H-TT provided the conception and design of research. WW, WH, W-BH, Y-LL, YT, H-MY, Q-JF, M-YZ, and Z-YW performed the experiments; WW and WH analyzed the data and interpreted the results of experiments; WW prepared the figures; WW and WH drafted the manuscript; Y-GW, H-TT, and R-MT edited and revised the manuscript; WW and Y-GW approved the final version of manuscript.

### Conflict of interest statement

R-MT and H-TT are employed by Suzhong Pharmaceutical Group Co., Ltd. The other authors declare that the research was conducted in the absence of any commercial or financial relationships that could be construed as a potential conflict of interest. The reviewer YS and handling Editor declared their shared affiliation.

## Abbreviations

Akt: serine-threonine kinase
Alb: serum albumin
ALT: alanine transaminase
α-SMA: α-smooth muscle actin
AM: *abelmoschus manihot* (L.) medic
AST: aspartate transaminase
BG: blood glucose
BUN: blood urea nitrogen
BW: body weight
CKD: chronic kidney disease
Col-I: collagen type I
DMSO: dimethylsulfoxide
DN: diabetic nephropathy
ECM: extracellular matrix
EM: electron microscopy
FN: fibronectin
4EBP1: eukaryotic initiation factor 4E-binding protein-1
GAPDH: glyceraldehyde-3-phosphate dehydrogenase
GBM: glomerular basement membrane
GCP: glomerular cellular population
GV: glomerular volume
HFD: high-fat diet
HG: high-glucose
HKC: Huangkui capsule
HRP: horseradish peroxidase
HYP: hyperoside
HPLC: high performance liquid chromatography
IF: immunofluorescence
KHI: kidney hypertrophy index
KW: kidney weight
LM: light microscopy
MCs: mesangial cells
micro-UAlb: micro-urinary albumin
MNT: mannitol
mTOR: mammalian target of rapamycin
NLRC3: NOD-like receptor family CARD domain containing 3
p70S6K: 70-kDa ribosomal protein S6 kinase
PAS: periodic acid-Schiff
PB: phosphate buffer
PI: protease inhibitors
PI3K: phosphatidylinositol-3-kinase
RAP: rapamycin
Scr: serum creatinine
SDS: sodium dodecyl sulfate
SDS-PAGE: sodium dodecyl sulfate-polyacrylamide gel electrophoresis
STZ: streptozotocin
TGF-β1: transforming growth factor-β1
WB: Western blot.
